# Prognostic Significance of the Tumor-Stroma Ratio in Epithelial Ovarian Cancer

**DOI:** 10.1155/2015/589301

**Published:** 2015-11-01

**Authors:** Ying Chen, Lei Zhang, Wenxin Liu, Xiangyu Liu

**Affiliations:** ^1^Department of Gynecologic Oncology, Tianjin Medical University Cancer Institute and Hospital, Tianjin 300060, China; ^2^Key Laboratory of Cancer Prevention and Therapy, Tianjin 300060, China; ^3^National Clinical Research Centre of Cancer, Tianjin 300060, China

## Abstract

Tumor-stroma ratio (TSR) has recently been identified as a promising prognostic parameter for several solid tumors. This study aimed to evaluate the prognostic role of TSR in epithelial ovarian cancer (EOC) and 838 EOC patients were enrolled in this study. TSR was estimated on hematoxylin-and-eosin-stained tissue sections from the most invasive part of the primary tumor. Patients were classified as stroma-rich or stroma-poor according to the proportion of stroma ≥50% or <50%. Chi-square test analysis revealed that TSR were significantly associated with FIGO stage, LN status, and recurrence or not (all of them *P* < 0.001). The higher stroma-rich proportions were found in EOC patients with advanced stage (36.13% versus 19.75%), LN metastasis (51.93% versus 27.25%), and recurrence (34.27% versus 6.82%). Stroma-rich EOC patients had obvious shorter median time of progression-free survival (29 versus 39 months) and overall survival (50 versus 58 months), respectively. TSR was an independent prognostic factor for the evaluation of PFS in EOC. Stroma-rich tumors had worse prognosis and higher risk of relapse compared with those in stroma-poor tumors in EOC patients. Considered easy to determine for routine pathological examination, TSR may serve as a new prognostic histological parameter in EOC.

## 1. Introduction

Approximately, 95% of ovarian cancers are of epithelial origin. In 2014, there were 21,980 estimated new diagnoses of ovarian cancer and 14,270 deaths from the disease, which is the most common cause of death among women with gynecologic cancer [1]. Tumor recurrence and metastasis are the leading cause of morbidity and mortality in EOC [2]. Traditionally, surgical pathological staging systems (International Federation of Gynecology and Obstetrics staging, FIGO staging) are still the most important tool for therapeutic decision-making in epithelial ovarian cancer (EOC). However, tumor cell pathological variables are only moderate indicators of outcome and therapy response. Since currently applied predictive factors do not adequately stratify risk in EOC patients, additional information is needed to individualize treatment [2].

Tumor invasion and metastasis are considered to be a multifactor process involving complex interactions of biological pathways [3]. The stroma surrounding cancer cells influences tumor development and behavior and the components of the tumor stroma have drawn increasing attention in predicting tumor prognosis [4]. Recently, as a consequence of the growing interest in the microenvironment, several studies have been conducted to evaluate the ratio of tumor to stroma (TSR) as a reflection of the microenvironment of cancer and survival outcome in esophageal cancer [5], breast cancer [6], colon cancer [7], and cervical cancer [8].

To our knowledge, the prognostic value of TSR has not been explored for EOC. Therefore, the objective of this study was to evaluate the prognostic value of TSR in EOC and its relationship with other prognostic factors.

## 2. Materials and Methods

### 2.1. Ethics Statement

Institutional review board approval was obtained for this study, and informed patient consent was waived owing to the retrospective nature of the study.

### 2.2. Patient Population

In this retrospective study, 1065 patients diagnosed and treated for EOC between January 2001 and December 2011 at the Department of Gynecologic Oncology, Tianjin Medical University Cancer Institute and Hospital, were enrolled. Sixty-two patients were excluded with a previous history of cancer (ten patients with breast cancer, fifteen with colon cancer, twelve with rectum cancer, and twenty-five with other cancers). Due to the known effect of neoadjuvant therapy on stromal formation in tissue, we excluded 165 patients receiving neoadjuvant chemotherapy, which could interfere with the evaluation of TSR. Thus, 838 patients were enrolled for further analysis and, among them, 806 samples received adjuvant treatment after surgery.

Age, FIGO stage, histologic subtype, histologic grade, residual tumors, CA125, ascites volume, lymph node state, events of recurrences, and patient status at follow-up were extracted from available follow-up records.

### 2.3. Histopathological Scoring

Tissue samples consisting of 4 *μ*m haematoxylin-and-eosin- (H&E-) stained sections from the most invasive part of the primary tumor were used for analysis using conventional microscopy. For TSR scoring, 2 investigators (Runfen Cheng and Yan Sun) estimated the TSR on all tumor slides and scored slides to the nearest 10 percentage points in a blinded manner. The most invasive tumor area of each slide was selected with the use of a 5x objective. The investigator chose a part of the sample containing both tumor and stromal tissue by using a 10x objective. Tumor cells had to be present at all borders of the image field. Mucinous tissue was visually excluded for scoring. For statistical analysis, the TSR was determined at the maximum discriminative power.

The tumor was evaluated per tenfold percentage (10, 20, 30%, etc.), and TSR values of 10% and 100% were not seen. In case of an inconclusive score, a third observer was consulted. In case of tumor heterogeneity, areas with the lowest TSR value were considered decisive as is performed in routine pathology to determine tumor differentiation.

### 2.4. Follow-Up

Follow-up data were collected until death or December 2014. All patients had a regular follow-up schedule including a complete history, serum tumor marker detection, physical examination, and routine imaging evaluation every 3 months during the first 2 years since the last time of treatment and every 6 months thereafter. Overall survival (OS) was defined as the time interval from the date of primary surgery to the date of death (failure) or to the end of follow-up for women who were alive (censored). Progression-free survival (PFS) was defined as the time elapsed from the date of primary surgery to the appearance of disease recurrence or progression (failure) or the last follow-up for women who were alive with no evidence of disease recurrence or progression (censored).

### 2.5. Statistical Analysis

For statistical analysis, SPSS (Statistical Package for the Social Sciences) version 18.0 (Chicago, IL, USA) was applied. The results were considered statistically significant with a probability of less than 0.05. The chi-squared and Fisher's exact tests were applied in analysis of categorical variable. The survival was determined by the Kaplan-Meier method, and the log rank test was used to determine significance. Factors that were deemed of potential importance by univariate analysis were included in the multivariate analysis by using Cox proportional hazard regression models. The prognostic significance of the TSR in EOC is also demonstrated by the subgroup analysis using the Cox proportional hazard regression model. Associations are shown as hazard ratios (HR) and 95% confidence intervals (CI).

## 3. Results

### 3.1. Patient Characteristics and Demographics

Clinicopathological characteristics of patients were shown in Table 1. In this study, a total of 838 patients with EOC were recruited between 2001 and 2011, and, among these patients, 207 cases have not received pelvic or para-aortic and pelvic lymphadenectomy owing to these patients with residual tumors ≥1 cm. The median age of the 838 patients at the time of surgery was 55 years (range: 21–79 years) and the median follow-up time was 50 months (3–119 months).

### 3.2. The Optimal Cutoffs of TSR for EOC Patients

To determine the optimal cutoff of TSR, we analyzed the *P* value for PFS and OS at different cutoffs in the statistical analysis and got the 50% level as the best cutoff point with maximum discriminating power for further analysis. Therefore, all patients were classified as “stroma-rich” or “stroma-poor” according to the proportion of stroma ≥50% or <50%, respectively (Table 2).

### 3.3. Relationships between TSR and Clinicopathological Variables in EOC Patients

**Figure 1:**
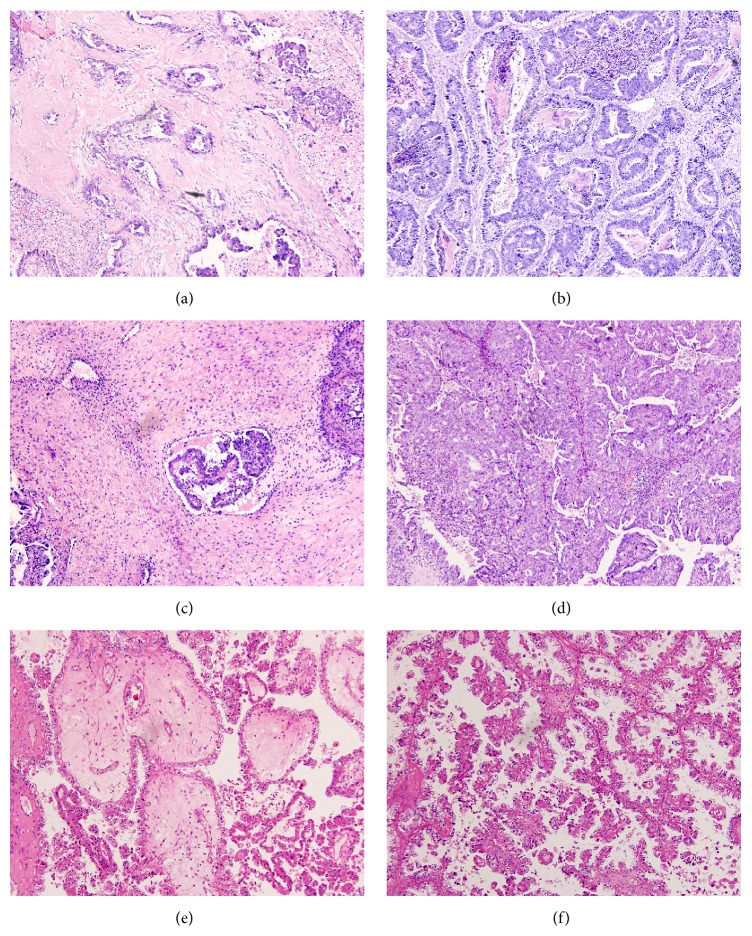
Representative images showing hematoxylin-and-eosin-stained 4 *μ*m sections of epithelial ovarian cancer (original magnification ×100). (a) Serous ovarian adenocarcinoma (G_2_) of stroma-rich type (TSR ≥ 50%). (b) Serous ovarian adenocarcinoma (G_1_) staining of stroma-poor type (TSR < 50%). (c) Serous ovarian adenocarcinoma (G_3_) of stroma-rich type (TSR ≥ 50%). (d) Serous ovarian adenocarcinoma (G_3_) of stroma-poor type (TSR < 50%). (e) Clear cell ovarian adenocarcinoma of stroma-rich type (TSR ≥ 50%). (f) Clear cell ovarian adenocarcinoma of stroma-poor type (TSR < 50%).

As shown in Table 3, we divided the patients into two groups, stroma-rich group (TSR ≥ 50%) and stroma-poor group (TSR < 50%), and compared the difference of clinicopathological characteristics between the two groups (Figure 1). Chi-square test analysis revealed that there were no significant differences between the stroma-rich and stroma-poor groups regarding patient age, menopausal status, tumor histology, residual disease, serum CA125 level, and ascites volume. However, the proportions of stroma were significantly associated with FIGO stage, LN status, and recurrence or not. The higher stroma-rich proportions were found in EOC patients with advanced stage (36.13% versus 19.75%), LN metastasis (51.93% versus 27.25%), and recurrence (34.27% versus 6.82%).

### 3.4. Survival and Multivariate Analysis

By the Kaplan-Meier method of univariate analysis, the shorter median of OS and PFS was related to advanced stage, LN metastasis, ascites volume >1000 mL, and stoma-rich type (all of them: *P* < 0.05, Table 4). Furthermore, shorter median of PFS was also related to low differentiation (*P* = 0.001, Table 4) and shorter median of OS was related to serum CA125 > 675 U/mL (*P* = 0.010, Table 4).

These significant variables detected by univariate analysis were included in multivariate analysis. In Cox proportional hazard model, advanced stage and stroma-rich type were the independent factors for the evaluation of PFS (*P* < 0.05, Table 4). Additionally, advanced stage and LN metastasis were the independent factors for the evaluation of OS (*P* < 0.05, Table 4).

Moreover, in the different subgroup analysis, according to the histology (serous and nonserous), FIGO stage (I-II and III-IV), LN status (metastasis and nonmetastasis), and residual disease (<1 and ≥1 cm), TSR also was identified as the significant indicator for PFS and OS by using Cox univariate proportional hazard regression model (Table 5).

**Figure 2:**
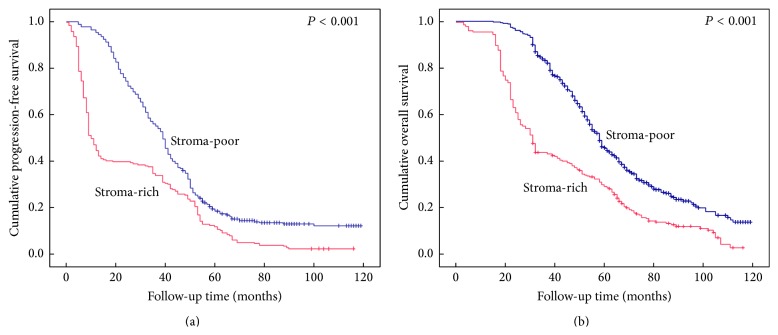
Kaplan-Meier curves for survival of 838 patients with epithelial ovarian cancer. Cumulative progression-free survival (a) and overall survival (b).

Generally, EOC patients with stroma-rich condition showed shorter PFS and OS than patients with stroma-poor condition. Kaplan-Meier survival curves are displayed in Figures 2(a) and 2(b).

## 4. Discussion

In the last decades, tumor cells have drawn the attention of the researchers as the main target for therapeutic interventions. However, evidence is growing that the peritumoral microenvironment plays key roles in tumor establishment and tumor cell dissemination [9]. The mechanisms of the tumor-stroma interaction are critical in tumor progression, offering significant therapeutic implications [10]. TSR was first reported as an independent factor for survival in colon cancer [7]. In addition to being a newly identified prognostic factor, it was also confirmed to be significantly associated with prognosis of esophageal squamous cell carcinoma [5], breast cancer [6], and cervical cancer [8].

However, its prognostic role in EOC is largely unknown although TSR is a convenient and useful tool for pathologists to obtain more prognostic information from H&E-stained slide. In this present study, we analyzed the prognostic value of TSR in 838 EOC patients. The optimal threshold level of TSR was determined on the basis of a maximum discriminating power for PFS and OS. We determined that 50% cutoff value was the most representative by use of statistical analysis. Therefore, all patients were classified as “stroma-rich” or “stroma-poor” according to the proportion of stroma ≥50% or <50%, respectively. The higher stroma-rich proportions were found in EOC patients with advanced stage, LN metastasis, and recurrence.

Tumor tissue is composed of both carcinoma cells and stromal cells recruited from normal tissue. Nowadays, accumulated lines of evidence had illustrated that, in normal tissue, the stroma may actually act as a barrier in tumorigenesis by constraining tumor cell proliferation [11, 12]. In tumor tissue, however, stromal components, the main part of tumor microenvironment, could facilitate the process of tumor progression [13]. The mechanism underlying tumor-promoting effect of stroma is still not fully understood. Previous evidence supports the notion that the increase in abundance of fibroblasts in tumor causes deposition of fibrotic extracellular matrix (ECM). Changes in ECM structure can be further stimulated by proteases, which degrade stroma. Together, this results in disruption of epithelial tissue and remodeling of the ECM, facilitating invasion of tumors cells [14].

Recently, the so-called cancer-associated fibroblasts (CAFs) composed of the major cellular components of tumor stroma caused more and more attention, which were found to have a predominant role in tumor growth and progression [15]. The CAF-derived regulators and extracellular matrix proteins can support cancer progression by providing a protective microenvironment for the cancer cells via reduction of chemotherapy sensitivity [16]. CAFs are frequently observed in the stroma of human carcinoma and secrete a variety of soluble factors such as transforming growth factor beta 1 (TGF-*β*1) [17], stromal cell-derived factor 1, and other soluble factors, which act in a paracrine manner and affect not only cancer cells but also other cell types present in the stroma [18]. As a sign of their activation, CAFs produce several mesenchyme-specific proteins such as fibroblast-specific protein (FSP-1), fibroblast-activating protein (FAP), vimentin, and alpha-smooth muscle actin (*α*-SMA), the prototypical marker for myofibroblasts. CAFs are also a rich source of different secreted factors such as cytokines and chemokines (e.g., IL-6, CXCL8, and CXCL12) and growth factors like epidermal growth factor (EGF) and vascular endothelial-derived growth factor (VEGF), which could promote angiogenesis, which is essential for tumor growth and progression [19, 20]. Additionally, CAFs have recently been investigated for their function as a regulator of immune cell recruitment and function [21]. Previous studies suggest CAFs are first educated by immune cells during the initial stages of tumorigenesis but they acquire the ability to recruit and regulate immune cells to an eventually immune-suppressed phenotype that is compatible with disease progression [22]. Moreover, a recent study by Herrera and coworkers investigating the role of CAFs in colon cancer suggested that the combination of CAFs and M2 macrophage signatures correlated with a clear difference in disease progression and survival of advanced stage patients [23].

Significantly, our results manifested the fact that EOC patients with stroma-rich condition had obvious shorter PFS and OS. Importantly, we revealed that TSR may be an independent and strong prognostic factor for the evaluation of PFS in EOC patients. However, our study has its shortcomings, which was retrospective study. Moreover, the mechanism underlying tumor-promoting effect of tumor in EOC was still not explored by us. It is of greater value to conduct a prospective study and investigate the molecular mechanism, which may avail us to determine whether the TSR could be used in clinical practice for better risk classification of EOC patients and even for implementation in standard pathology reports in the future.

Conclusively, our findings indicate EOC patients could be classified as “stroma-rich” or “stroma-poor” according to the best cutoff of TSR 50%. The higher stroma-rich proportions were found in EOC patients with advanced stage, LN metastasis, and recurrence. TSR may be an independent and strong prognostic factor for the evaluation of PFS in EOC patients. The TSR is easy to determine, reproducible, and quickly performed by using routine pathological examination on H&E-stained sections. Thus, the study of tumor stroma has potential to facilitate the prognostic assessment of EOC patients in the clinical practice and even in combination with other therapeutic agents for individual treatment in the future.

## Figures and Tables

**Table 1:** Clinicopathologic characteristics, demographics, and values for 838 EOC patients.

Age (years)	Median: 55; range: 21–79
BMI (kg/m^2^)	Median: 23; range: 15–47
Menopausal status	
Yes	505 (60.3%)
No	333 (39.7%)
Histology	
Serous	599 (71.5%)
Mucous and others	231 (28.5%)
Differentiation	
G_1-2_	420 (50.1%)
G_3_	418 (49.9%)
FIGO stage (2009)	
I-II	243 (29.0%)
III-IV	595 (71.0%)
With lymphadenectomy	631 (75.2%)
Pelvic	527 (83.5%)
Para-aortic + pelvic	104 (16.5%)
Removed lymph nodes	Median: 25; range: 12–57
Lymph nodes metastasis	
No	642 (76.6%)
Yes	196 (23.4%)
Residual disease	
<1 cm	631 (75.3%)
≥1 cm	207 (24.7%)
Ascites volume (mL)	Median: 1000; range: 0–7000
≤1000	591 (70.5%)
>1000	247 (29.5%)
Serum CA125 (U/mL)	Median: 675; range: 23–7400
≤675	521 (62.2%)
>675	317 (37.8%)
Tumor-stroma ratio (TSR)	
Stroma-poor (TSR < 50%)	575 (68.6%)
Stroma-rich (TSR ≥ 50%)	263 (31.4%)

**Table 2:** The determination of the best cutoff for tumor-stroma ratio (TSR) in EOC.

TSR	Number	3-year PFS	*χ* ^2^	*P*	5-year survival rate	*χ* ^2^	*P*
<20%	6	0	0.671	0.413	0	0.289	0.591
≥20%	832	48.2%			40%		

<30%	91	52.5%	3.240	0.072	47.2%	4.312	0.038
≥30%	747	47.6%			38.1%		

<40%	221	58.7%	2.402	0.087	48.4%	5.605	0.018
≥40%	617	54.2%			37.1%		

<50%	575	55.3%	13.704	**<0.001**	45.6%	12.251	**<0.001**
≥50%	263	33.8%			29.1%		

<60%	665	46.5%	3.965	0.046	46.6%	7.338	0.007
≥60%	173	42.2%			34.4%		

<70%	717	47.8%	3.058	0.080	40.2%	3.778	0.052
≥70%	125	52.8%			41.4%		

<80%	796	47.5%	0.399	0.528	39.8%	1.611	0.204
≥80%	42	69%			53.2%		

<90%	824	48.4%	0.103	0.749	40.3%	0.992	0.319
≥90%	14	57.1%			47.6%		

PFS: progression-free survival.

Analysis was performed using the Kaplan-Meier method.

The minimum *P* value was indicated in bold font.

**Table 3:** Relationships between TSR and characteristics in 838 EOC patients.

Characteristics	Total	Stroma-poor	Stroma-rich	Stroma-rich proportion	*P*
	(*N* = 838)	(*N* = 575)	(*N* = 263)	(*N* = 263)	
	Number (%)	Number (%)	Number (%)	Number (%)	
Age, year					0.365
≤55	347 (41.41)	232 (40.35)	115 (43.73)	115 (33.14)	
>55	491 (58.59)	343 (59.65)	148 (56.27)	148 (30.14)	
Menopausal status					0.820
Yes	505 (60.26)	348 (60.52)	157 (59.70)	157 (31.09)	
No	333 (39.74)	227 (39.48)	106 (40.30)	106 (31.83)	
Histology					0.805
Serous	599 (71.48)	409 (71.13)	190 (72.24)	190 (31.72)	
Mucous and others	239 (28.52)	166 (28.87)	73 (27.76)	73 (30.54)	
Differentiation					0.070
G_1-2_	420 (50.12)	276 (48.00)	144 (54.75)	144 (34.29)	
G_3_	418 (49.88)	299 (52.00)	119 (50.42)	119 (28.47)	
FIGO stage (2009)					<0.001
I-II	243 (29.00)	195 (33.91)	48 (18.25)	48 (19.75)	
III-IV	595 (71.00)	380 (66.09)	215 (81.75)	215 (36.13)	
Lymph nodes metastasis					<0.001
No	657 (78.40)	478 (83.13)	179 (68.06)	179 (27.25)	
Yes	181 (21.60)	87 (16.87)	94 (31.94)	94 (51.93)	
Residual disease					0.796
<1 cm	631 (75.30)	431 (74.96)	200 (76.05)	200 (31.70)	
≥1 cm	207 (24.70)	144 (25.04)	63 (23.95)	63 (30.43)	
Ascites volume (mL)					0.464
≤1000	591 (70.53)	410 (71.30)	181 (68.82)	181 (30.63)	
>1000	247 (29.47)	165 (28.70)	82 (31.18)	82 (33.20)	
Serum CA125 (U/mL)					0.091
≤675	521 (62.17)	346 (60.17)	175 (66.54)	175 (33.59)	
>675	317 (37.83)	229 (39.83)	88 (33.46)	88 (27.76)	
Recurrence					<0.001
No	88 (10.50)	82 (14.26)	6 (2.28)	6 (6.82)	
Yes	750 (89.50)	493 (85.74)	257 (97.72)	257 (34.27)	

**Table 4:** Univariate and multivariate survival analysis of the prognostic factors for progression-free and overall survival in 838 EOC patients.

Variable	Cases (*N*)	TR cases (*N*)	TD cases (*N*)	Progression-free survival (PFS)					Overall survival (OS)				
				Univariate analysis		Multivariate analysis			Univariate analysis		Multivariate analysis		
				Median of PFS	*P* ^a^	HR	95% CI for HR	*P* ^b^	Median of OS	*P* ^a^	HR	95% CI for HR	*P* ^b^
Age (year)					0.260	NA	NA	NA		0.061	NA	NA	NA
≤55	347	316	251	40					58				
>55	491	434	359	31					48				
Menopausal status					0.564	NA	NA	NA		0.076	NA	NA	NA
Yes	505	449	380	32					49				
No	333	301	230	39					58				
Histology					0.576	NA	NA	NA		0.123	NA	NA	NA
Serous	599	533	422	35					54				
Mucous and others	239	217	188	37					53				
Differentiation					0.001	1.142	0.979–1.333	0.092		0.060	NA	NA	NA
G_1-2_	420	368	311	40					59				
G_3_	418	382	299	32					50				
FIGO stage (2009)					<0.001	0.773	0.657–0.908	0.002		0.001	0.710	0.612–0.824	<0.001
I-II	243	210	155	42					58				
III-IV	595	540	455	30					49				
LN metastasis					0.020	0.869	0.732–1.031	0.108		<0.001	0.774	0.659–0.911	0.002
No	657	572	451	37					57				
Yes	181	178	159	32					48				
Residual disease					0.764	NA	NA	NA		0.716	NA	NA	NA
<1 cm	631	561	451	36					55				
≥1 cm	207	189	159	34					50				
Ascites volume (mL)					0.047	0.929	0.785–1.100	0.393		0.002	0.922	0.778–1.092	0.345
≤1000	591	525	414	36					55				
>1000	247	225	196	34					51				
Serum CA125 (U/mL)					0.154	NA	NA	NA		0.010	1.149	0.984–1.341	0.079
≤675	521	458	359	35					55				
>675	317	292	251	35					52				
STR					<0.001	0.731	0.615–0.827	<0.001		0.001	1.172	0.983–1.396	0.076
Stroma-poor	575	493	381	39					58				
Stroma-rich	263	257	229	29					50				

TR: tumor recurrence; TD: tumor-related death; LN: lymph node; *P* ^a^: *P* value, log rank test; HR: hazard ratio; CI: confidence interval; *P* ^b^: *P* value, Cox regression; NA: not applicable.

**Table 5:** The significance of TSR in 838 EOC patients according to different subgroup using multivariate Cox survival analysis.

Variable	Cases (*N*)	TR (*N*)	TD (*N*)	TSR					
				Progression-free survival (PFS)			Overall survival (OS)		
				HR	95% CI	*P*	HR	95% CI	*P*
Histology									
Serous	599	533	422	1.873	1.564–2.242	<0.001	2.075	1.702–2.530	<0.001
Mucous and others	239	217	188	1.616	1.210–2.157	0.001	1.860	1.348–2.567	<0.001
FIGO stage (2009)									
I-II	243	210	155	1.065	0.765–1.481	0.710	1.038	0.705–1.528	0.849
III-IV	595	540	455	2.058	1.726–2.455	<0.001	2.302	1.904–2.783	<0.001
LN metastasis									
No	657	572	451	1.276	1.082–1.505	0.004	1.818	1.492–2.216	<0.001
Yes	181	178	159	2.156	1.588–2.926	<0.001	2.555	1904–2.783	<0.001
Residual disease									
<1 cm	631	561	451	1.961	1.646–2.337	<0.001	2.167	1.790–2.624	<0.001
≥1 cm	207	189	159	1.387	1.015–1.896	0.040	1.607	1.135–2.276	0.007

TSR: tumor-stroma ratio; TR: tumor recurrence; TD: tumor-related death; LN: lymph node; HR: hazard ratio; CI: confidence interval; *P*: *P* value, Cox regression.
